# Association Between Sarcopenic Obesity and Frailty Risk in Community-Dwelling Older Women With Locomotive Syndrome: A Cross-Sectional Survey

**DOI:** 10.7759/cureus.64612

**Published:** 2024-07-15

**Authors:** Naoki Deguchi, Ryo Tanaka, Tomoyuki Akita

**Affiliations:** 1 Geriatrics, Tokyo Metropolitan Institute for Geriatrics and Gerontology, Tokyo, JPN; 2 Graduate School of Humanities and Social Sciences, Hiroshima University, Hiroshima, JPN; 3 Graduate School of Biomedical and Health Sciences, Hiroshima University, Hiroshima, JPN

**Keywords:** frailty phenotype, obesity, sarcopenia, body fat, skeletal muscle mass

## Abstract

Background

Concurrent sarcopenia and obesity in locomotive syndrome (LS) impair activities of daily living and decrease extremity muscle strength and motor function. However, the increased risk of frailty posed by sarcopenic obesity compared to either sarcopenia or obesity alone remains unclear.

Objective

To examine the association between sarcopenic obesity and frailty risk in community-dwelling older adult women with LS.

Methods

This cross-sectional study included 158 women aged ≥65 years with LS stage 1 (age, 74.0 yrs, body mass index, 22.7 kg/m^2^) according to the Japanese Orthopaedic Association criteria. Bioelectrical impedance analysis was used to measure the skeletal muscle mass index (SMI) and percent body fat (PBF). Participants were classified into four subtypes: normal (non-obesity, non-sarcopenia), sarcopenia (SMI < 5.7 kg/m^2^), obesity (PBF > 35%), and sarcopenic obesity (SMI < 5.7 kg/m^2^ and PBF > 35%). Logistic regression analysis was used to adjust for age, body mass index, back pain, knee pain, history of falls, and physical function.

Results

Among the participants, 52 individuals (32.9%) were classified as frailty risk. The percentage of body phenotypes was 30.4% normal, 32.9% were sarcopenia, 22.8% had obesity (RT1), and 13.9% had sarcopenic obesity. The odds ratios for frailty risk compared to normal were 3.97 (95% confidence interval (CI): 1.51 to 10.4), 1.71 (95% CI: 0.55 to 5.39), and 4.25 (95% CI: 1.34 to 13.5) for sarcopenia (RT2), obesity, and sarcopenic obesity subtypes, respectively, sarcopenia and sarcopenic obesity were significantly associated with frailty risk.

Conclusion

In older adult women with LS, the presence of sarcopenia or sarcopenic obesity may increase the risk of frailty; however, the addition of obesity does not always further increase this risk. Further investigation of the association between increased body fat and frailty in older adult women is warranted.

## Introduction

Frailty is characterized by diminished physiological reserve in the older adults, causing increased vulnerability to stress, which can lead to functional incapacity, increased dependency, and an increased risk of mortality [1,2]. The prevalence of frailty increases with age, subsequently increasing the likelihood of adverse outcomes such as falls, physical impairment, hospitalization, and death [3]. These findings demonstrate the importance of managing the stages before frailty (pre-frailty) to promote healthier aging.

Decreases in body fat [4,5] and skeletal muscle mass [6,7] have both been linked to an increased risk of frailty. In addition, recent studies [8,9] have elucidated the relationship between the loss of skeletal muscle mass and increased body fat with a decline in physical function, as well as the underlying mechanisms of their effects. Consequently, the combination of low muscle mass and excess body fat is a promising target for preventing frailty in older adults. In late midlife, the greater body fat among women predisposed them to higher frailty levels in late midlife into old age [10]. This finding demonstrates the importance of including women in body composition and frailty studies.

Locomotive syndrome (LS), introduced by the Japanese Orthopaedic Association (JOA), is a condition characterized by limited mobility due to musculoskeletal disorders [11]. LS is classified from stages 1 to 3 based on severity [11]. Only 2.4% of older adults in LS stage 1 are considered frail; however, all older adults with frailty are in LS stage 1, thus preventing LS is considered key to preventing frailty [12]. Previous studies have shown that the LS criteria overlap with frailty, with 90.1% of the frailty population meeting the LS criteria, suggesting that the LS criteria’s potential as the best tool for screening older adults at risk of long-term care in the future [13]. Additionally, older adults with LS stage 1 have significantly lower physical functioning at five years than those without LS stage 1. Stage 1 LS screening is important for the prevention of motor dysfunction in middle-aged and older adults [14]. Consequently, focusing on LS stage 1 allows for early identification and subsequent intervention before progression to frailty.

Research on body composition in LS suggests that the presence of both sarcopenia (characterized by decreased muscle mass and strength) and obesity in LS is associated with a further decline in the activities of daily living, lower limb muscle strength, and motor function [15-17]. Thus, it remains unclear whether low muscle mass or obesity is at greater risk for frailty in the older adult population with LS. This study focuses on older adult women with LS stage 1, classified into subtypes based on body composition (normal, sarcopenic, obesity, and sarcopenic obesity), and aims to investigate the associations between these subtypes and the risk of frailty.

## Materials and methods

Study design and participants

This cross-sectional study used integrated data from the “Study for Diagnosis, Early Detection, and Treatment of Locomotor Syndrome using Epidemiological Cohort (DETECt-L)” in 2023 (April to December). DETECt-L is a comprehensive longitudinal study of men and women living in Hiroshima, Higashi-Hiroshima, and Kure City. Participants for this study were recruited using convenience sampling; staff members at two sports and two regional centers or exercise instructors who gave their consent distributed flyers to recruit participants. Upon receiving the flyers, those who wished to participate gathered at the designated facility where their measurements would be taken.

Measurements were performed in a gymnasium or a room with at least 5 meters of walking distance. The measurements were performed by physical therapists and exercise instructors with experience in motor function testing. The inclusion and exclusion criteria were explained to the participants verbally and in writing prior to the start of the measurements, and participants who met the exclusion criteria were not included in the measurement sessions. The exclusion criteria of the DETECt-L were as follows: 1) no suspected cognitive impairment, and 2) absence of severe conditions wherein exercise is contraindicated, such as unstable cardiovascular disease, stroke, severe respiratory disease, Parkinson’s disease, diabetic peripheral neuropathy, and rheumatism or arthritis.

The DETECt-L included 334 adults aged ≥65 years. Of these, 158 who had no missing values and met the LS stage 1 diagnostic criteria for LS published by the JOA [11] (Table 1), namely, unable to stand up from a 40 cm platform on one leg but able to stand up from a 20 cm platform on both legs on the stand-up test, ≥1.1 but <1.3 on the 2-step test, or ≥7 points but <16 points on the geriatric locomotor function scale 25, were included in the analysis. Exclusion criteria were other stages than LS stage 1 (n = 129; non-LS: n = 73, LS stage 2: n = 33, and stage 3: n = 23). This study was conducted with the approval of the Ethics Review Committee of Hiroshima University (application number: E2022-0086). The research was performed in accordance with the Declaration of Helsinki.

**Table 1:** Diagnosis of locomotive syndrome GLFS; Geriatric Locomotive Function Scale

Stages	Diagnosis
Stage 1	1. Stand-up test: Difficulty in standing on one leg from a 40-cm chair
	2. Two-step test: ≥ 1.1 but less than < 1.3
	3. GLFS25: ≥7 points but <16 points
Stage 2	1. Stand-up test: Able to stand up with both feet from 40- and 30-cm chairs but difficult to stand up with both feet from a 20-cm chair
	2. Two-step test: ≥ 0.9 m and <1.1
	3. GLFS25: ≥16 points but < 24 points
Stage 3	1. Stand-up test: Able to stand up with both feet from a 40-m chair but unable to stand up with both feet from a 30-cm chair
	2. Two-step test: <0.9
	3. GLFS25: ≥24 points

Measurements

Frailty Phenotype

The Japanese version of the Cardiovascular Health Study (J-CHS) framework assesses frailty based on five criteria: muscle strength loss, slowing of walking speed, fatigue, reduction in physical activity, and weight loss [18]. Muscle strength decline is determined by measuring grip strength in both hands twice each using a hand dynamometer (Grip-D TKK5101, Takei Scientific Instruments Co., Ltd., Niigata, Japan), with the classification of muscle strength decline based on an average maximum value of <18 kg. Slowing of walking speed was defined as a speed <1.0 m/s during a 5-meter walk at an average pace. Fatigue was defined as a subjective feeling of being unreasonably tired in the past 2 weeks. Reduced physical activity was assessed based on the frequency of participation in activities, as well as regular exercise or sports at least once a week). Weight loss was determined by a decrease in weight of more than 2 kg in the last six months. According to these J-CHS criteria, individuals who meet ≥3 of the specified conditions are classified as “frail,” those who meet 1-2 of the criteria are classified as “pre-frail,” and those who meet none of the conditions are classified as “robust.” In this study, participants were divided into “robust” and “pre-frail” groups, and frailty risk was defined as pre-frailty or above.

Sarcopenia, Obesity, and Sarcopenic Obesity

The assessment of sarcopenia and obesity was performed using the InBody 270 (InBody Japan Co., Ltd., Tokyo, Japan), which uses bioelectrical impedance analysis to measure skeletal muscle mass index (SMI) and body fat percentage. The skeletal muscle mass was converted into SMI by dividing the muscle mass by the square of the participant’s height (kg/m^2^). If skeletal muscle mass was lower than the cut-off value (<5.7 kg/m^2^), the participants were considered as having low muscle mass (Sarcopenia) [19]. Obesity was diagnosed based on the National Health and Nutrition Examination Surveys, with a body fat percentage cut-off of ≥35% [20].

**Figure 1:**
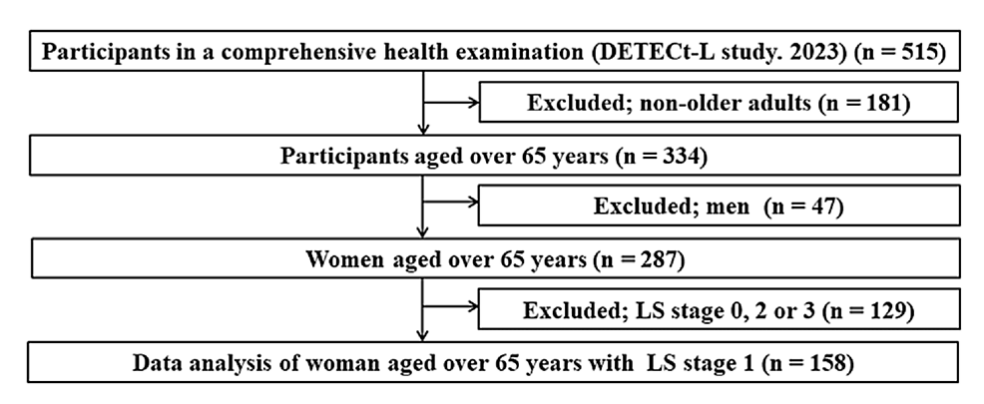
Participant flow DETEC-L study; Study for Diagnosis, Early Detection, and Treatment of Locomotor Syndrome using Epidemiological Cohort, LS; locomotive syndrome

Subtypes were categorized into four groups based on SMI and body fat percentage: normal (non-obesity and non-sarcopenia), obesity (obesity and non-sarcopenia), sarcopenia (non-obesity and sarcopenia), and sarcopenic obesity (obesity and sarcopenic) (Figure 1).

Covariates

This study investigated multiple factors associated with health assessments in older adults, such as demographic elements (age and body mass index (BMI), aspects of pain (knee and lower back pain), and history of falls using questionnaires and performance tests (timed up-and-go (TUG) test and single-leg standing time with eyes open).

A verbal rating scale was used to gauge the frequency of pain experienced during knee pain of walking and current low back pain, categorizing it into three distinct scales: “none,” “occasional,” and “constant.” We further streamlined these categories into a binary classification of either “none” or “occasional/constant.” The reliability and validity of this scale have been established for older adults with cognitive impairment [21].

Regarding fall history, participants were queried using a three-point scale based on the number of falls in the past year (0, 1, and ≥2 falls). For our study, these data were categorized into those with no falls and those with at least one fall [22]. During the TUG test [23], participants were timed as they rapidly rose from a chair with a backrest, walked three meters, returned, and then sat down again, measured twice, with the fastest value used.

For the one-leg standing time with eyes open, the participants selected either the left or right lower limb as the supporting leg, which was assessed by instructing participants. The maximum number of measurements was two, with an upper limit of 60 seconds, and the longer of the two measurements was used [24].

Statistical analysis

This study categorized body composition into four subtypes: normal, sarcopenia, obesity, and sarcopenic obesity. Continuous variables were expressed as median and interquartile range (IQR), while categorical variables were expressed as percentages. Multinomial logistic regression analysis was applied using frailty risk (robust/pre-frailty or frailty) and body phenotype (normal; non-sarcopenia/non-obesity as a reference, sarcopenia; sarcopenia/non-obesity, obesity; non-sarcopenia/obesity, and sarcopenic obesity; sarcopenia/obesity) as explanatory variables and was adjusted for potential confounding factors. Model 1 was unadjusted, Model 2 was BMI, and Model 3 included variables such as age, BMI, knee pain, back pain, falls, TUG, and one-legged standing in the adjusted model. All analyses were performed using SPSS software, version 28.0 (IBM Corp, Armonk, NY, USA). Statistical tests were two-tailed, with a significance level of α = 0.05.

## Results

Participant characteristics are shown in Table 2. The median age was 74.0 years, and the BMI was 22.7 kg/m2. The percentage of patients with pain was 45.6% for knee pain and 57.0% for lower back pain. The distribution of the frailty phenotype was dominated by robust individuals, representing 67.1% of the population. Conversely, frail subjects were markedly rare, representing only 1.3% of the population. Most individuals at frailty risk (pre-frail or frailty) were in a pre-frailty state. The distribution of subtypes was as follows: normal 30.4%; sarcopenia 32.9%; obesity 22.8%; and sarcopenic obesity 13.9%.

**Table 2:** Characteristics of the participants Quantitative variables are presented in the median and interquartile range. LS: Locomotive syndrome, GLFS-25: 25-question Geriatric Locomotive Function Scale.

Parameters	
Age, yrs	74.0 (70.0 – 79.0)
Body mass index, kg/m^2^	22.7 (20.7 – 24.6)
Knee pain, yes, n (%)	72 (45.6)
Low back pain, yes, n (%)	90 (57.0)
Timed up and go test, sec	5.6 (5.1 – 6.2)
One leg standing, sec	36.8 (13.8 – 60.0)
Fall history, yes, n (%)	28 (17.7)
Skeletal muscle index, kg/m^2^	5.7 (5.3 – 6.1)
Fat mass, %	32.8 (28.7 – 37.2)
LS stage 1 category, n (%)	
Two-step test	64 (34.2)
Stand-up test	146 (78.1)
GLFS-25	101 (54.0)
Frailty phenotype, (%)	
Robust	106 (67.1)
Pre-frailty	50 (31.6)
Frailty	2 (1.3)
Body phenotype, n (%)	
Normal	48 (30.4)
Sarcopenia	52 (32.9)
Obesity	36 (22.8)
Sarcopenic obesity	22 (13.9)

Table 3 shows the results of logistic regression on the association of frailty risk (pre-frail or frail) with body phenotype. In the unadjusted model, compared to the group with a “normal” body phenotype, i.e., no sarcopenia or obesity, sarcopenia (3.97, 95% CI = 1.56 to 10.1) and sarcopenic obesity (5.00, 95%CI = 1.62 to 15.5) were significantly related to the risk of frailty (pre-frail or frail). These results were also significantly related to Model 2, which was adjusted for BMI, with similar odds ratios in sarcopenia (4.15, 95% CI = 1.61 to 10.7) and sarcopenic obesity (4.86, 95% CI = 1.57 to 15.1) to Model 1. In Model 3, adjusted for all confounders, odds ratios for sarcopenia (3.97, 95% CI = 1.51 to 10.4) and sarcopenic obesity (4.25, 95% CI = 1.34 to 13.5) were similar. However, for women with obesity, the risk of frailty was not significant in all models.

**Table 3:** Frailty risk and sarcopenic obesity in older adult women CI; confidence interval, Ref.; reference, ＊p<0.05, † p<0.01 Model 1: unadjusted, Model 2: sex, body mass index, Model 3: age, body mass index, knee pain, low back pain, fall, timed up and go, one leg standing

	Model 1	Model 2	Model 3
	Odds (95%CI)	Odds (95%CI)	Odds (95%CI)
Normal	1.00 (ref)	1.00 (ref)	1.00 (ref)
Sarcopenia	3.97 (1.56 to 10.1) ^†^	4.15 (1.61 to 10.7) ^†^	3.97 (1.51 to 10.4) ^†^
Obesity	1.92 (0.67 to 5.51)	1.65 (0.53 to 5.13)	1.71 (0.55 to 5.39)
Sarcopenic obesity	5.00 (1.62 to 15.5) ^†^	4.86 (1.57 to 15.1) ^†^	4.25 (1.34 to 13.5)^＊^

## Discussion

This was a cross-sectional investigation of the association of obesity, sarcopenia, or both with the risk of frailty in older adult women with LS stage 1. In older adult women with LS stage 1, a possible precursor to frailty, low muscle mass, and sarcopenic obesity were associated with a risk of pre-frailty or frailty, independent of confounding factors, such as BMI, pain, history of falls, balance function, and age. Sarcopenic obesity, characterized by decreased muscle mass and increased body fat, is a public health concern; however, in primary care, it is misdiagnosed as simple obesity and is generally untreated [25]. Our findings highlight the importance of interventions that focus on muscle and fat mass, regardless of BMI, in efforts to prevent frailty and disability in this population.

Previous studies have reported that sarcopenia and sarcopenic obesity are associated with an increased risk of frailty in community-dwelling older adults; however, their association with obesity alone has not been confirmed [26]. Similarly, sarcopenic obesity in community-dwelling older men has been associated with frailty but not with obesity alone and frailty [27]. These reports are consistent with the findings in the present study showing no association between obesity and frailty risk in LS stage 1 older adult women. These findings suggest that body phenotype and obesity in older adults may not be associated with frailty risk. Interestingly, previous studies in men have not found an association between sarcopenia and frailty [27]. Thus, the association between frailty and body composition differs by sex, and women may be at increased risk of frailty simply because of lower muscle mass.

In this study, the prevalence of frailty and pre-frailty was observed to be 1.3% and 31.6%, respectively. This prevalence of frailty is marginally lower than the 2.4% reported in a prior study concerning older adults with LS stage 1 [12]. Such a discrepancy can be attributed to the distinct characteristics of the sample population, including the recruitment of participants from health facilities and gender differences. Furthermore, the prevalence of pre-frailty has not been reported in previous studies, and the pre-frailty rate among older women with LS is a noteworthy finding.

This study had several limitations. First, only 1.3% of the study population had frailty, which is significantly lower than that reported in other cohort studies [12,13,28]. This discrepancy suggests a predominance of physically and mentally robust older individuals in our cohort. Second, although 158 women were surveyed in this study, there are limitations to the generalizability of the results because random sampling was not used. This limited sample size particularly affects the ability to detect rare events and increases the risk of misinterpretation, requiring careful interpretation for the generalizability of the results to a broad population. Finally, as a cross-sectional study, a causal relationship cannot be established between sarcopenic obesity and the risk of frailty. Future research, preferably longitudinal in nature, is essential to elucidate these causal relationships.

## Conclusions

Sarcopenia and sarcopenic obesity were associated with an increased risk of frailty independently of BMI compared with the normal body phenotype among older adult women with LS stage 1. However, obesity had no significant effect on frailty risk, and sarcopenia and sarcopenic obesity had similar odds of frailty risk. These conclusions emphasize the importance of focusing on the prevention and control of sarcopenia and sarcopenic obesity in the health management of the older adult, focusing not only on weight and BMI, but also on maintaining and improving muscle mass.
